# Identification and phylogenetic analysis of oral *Veillonella *species isolated from the saliva of Japanese children

**DOI:** 10.12688/f1000research.18506.5

**Published:** 2019-09-13

**Authors:** Ariadna A. Djais, Citra Fragrantia Theodorea, Izumi Mashima, Maiko Otomo, Masato Saitoh, Futoshi Nakazawa

**Affiliations:** 1 Department of Oral Biology, Faculty of Dentistry, Universitas Indonesia, Jakarta, 10430, Indonesia; 2 Department of Oral Microbiology, School of Dentistry, Health Sciences University of Hokkaido, Ishikari-Tobetsu, Hokkaido, 061-0293, Japan; 3 Department of Microbiology, School of Pharmacy, Aichi Gakuin University, Kusumoto-cho, Nagoya, 470-0915, Japan; 4 Department of Pediatric Dentistry, School of Dentistry, Health Sciences University of Hokkaido, Ishikari-Tobetsu, Hokkaido, 061-0293, Japan

**Keywords:** Oral Veillonella species, dental caries, oral hygiene status, indicator, phylogenetic, saliva, children, Japan

## Abstract

**Background: **As the most frequent infectious disease among children worldwide, dental caries have a strong relationship with oral hygiene status, specifically in the development of infection. However, the study regarding the identification and distribution of oral *Veillonella *are limited. The oral *Veillonella *community may affected by the differences in geographical location, age, diet, lifestyle, socio-economic status and oral hygiene status. Here, we studied the oral hygiene status by examining the composition and proportion of oral *Veillonella* species in saliva of Japanese children.

**Methods:** Microbial samples collected from 15 Japanese children divided into three oral hygiene groups were cultured under anaerobic conditions after homogenization and dilution, and inoculated onto brain heart infusion and selective medium *Veillonella *agar. Genomic DNA was extracted from each isolate. *Veillonella* species were detected by one-step PCR using *rpoB* species-specific primers. To analyse the phylogenetic properties of the unknown *Veillonella* strains, PCR amplification and sequence analysis of *rpoB* were conducted for 10 representative strains.

**Results:** Although *V. rogosae *was found as the predominant species among all groups, its prevalence was significantly lower in the children with poor oral hygiene than in those with good oral hygiene. *V. parvula *was the prevalent species in the poor oral hygiene group. Approximately 10% of the isolated *Veillonella* strains were not classified to any established species; the phylogenetic analysis showed that they were most closely related to *V.* *infantium*

**Conclusions:** This study demonstrates that the composition and proportion of oral *Veillonella* species in the saliva of Japanese children is correlated with different oral hygiene status. Changes in detection ratios of *V. parvula* and *V. rogosae* can be useful indicators of oral hygiene status. Furthermore, new strains closely related to *V. infantium* were isolated from the saliva of Japanese children.

## Introduction

The oral biofilm comprises multiple bacterial species and develops as a result of adhesion of pioneer bacterial species to adsorption of salivary proteins and glycoproteins on the enamel surface. These biofilms are not formed by random simultaneous colonization, but rather by selective, reproducible, and sequential colonization ^1, 2^. Oral biofilms are a source of bacteria that cause oral infections, for instance dental caries and periodontal disease, and they sometimes lead to or worsen systemic diseases ^3^.

Saliva is an acknowledged pool of biological markers that range from biochemical molecules changes such as DNA, RNA, and proteins, to those in microbiota structural composition ^4^. Furthermore, saliva has an important role in oral biofilm development and maintenance. Recently, metagenomic analysis from saliva samples of Thai children demonstrated that *Streptococcus* and *Veillonella* were the predominant bacterial genera in the samples, and the proportion of *Streptococcus* decreased, while that of *Veillonella* increased in the children with poor oral hygiene status ^5^.

The genus *Veillonella* consist of multiple gram-negative bacterial species, obligate anaerobic, non-motile, non-spore forming, small cocci belonging to the family Veillonellaceae ^6^.

No *Veillonella* species ferment carbohydrates or amino acids, except for *V. criceti*, *V. ratti*, and *V. seminalis*. The metabolic end products of *Veillonella* species from trypticase-glucose-yeast extract are mainly acetic acid and propionic acid ^6^. *Veillonella* species are present as commensal organisms in the oral cavity, intestinal tract and genitouritary and respiratory systems of humans and animals. Previous studies have reported that *Veillonella* species are rare causative organisms of meningitis, endocarditis, bacteraemia, discitis, vertebral osteomyelitis, and prosthetic joint infection ^7– 9^. Generally, *Veillonella* species are known to be resistant to tetracycline and sensitive to penicillin and ampicillin. However, some *Veillonella* strains resistant to both penicillin and ampicillin have recently emerged ^10^.

There are 14 species reported to belong to genus *Veillonella* including *V. infantium* which was assign as a novel species in 2018 ^11^. Of the 14 documented species, *V. atypica*, *V. denticariosi*, *V. parvula*, *V. rogosae*, *V. dispar*, *V. infantium*, and *V. tobetsuensis* have been found in human saliva or on tongue or dental biofilms ^12– 17^. Periasamy and Kolenbrander reported that oral *Veillonella* species are an early colonizer during the formation of oral biofilm, along with *Streptococcus* species, which were reported as initial colonizers in developing multispecies communities of oral biofilm ^18^. Therefore, it is important to determine the role of oral *Veillonella* species in formation of oral biofilm to improve the prevention and treatment of oral infectious diseases.

*Veillonella* strains are relatively easy to identify at the genus level, but remain difficult to identify at the species level, since there are no useful phenotypic or biochemical examinations to distinguish them ^19^. To resolve this problem, Mashima *et al.* established a novel one-step PCR method with species-specific primer sets based on the variable region of the *rpoB* gene sequences of oral *Veillonella* species ^12^. Additionally, 1,442 *Veillonella* strains isolated from the saliva of 107 Thai children were identified by this method as *V. dispar*, *V. parvula*, *V. rogosae*, *V. atypica*, *V. denticariosi*, and *V. tobetsuensis* in our previous study ^20^. In that study, *V. parvula* was significantly more prevalent in the poor oral hygiene, and the detection rate of oral *Veillonella* species in the saliva was indicative of the oral hygiene status of Thai children ^20^. Additionally, another study suggested that several novel *Veillonella* species may inhabit the human oral cavity ^21^. However, the study regarding the identification and distribution of oral *Veillonella* are limited. The oral *Veillonella* community may affected by the differences in geographical location, age, diet, lifestyle, socio-economic status and oral hygiene status.

Therefore, in this study, we examined composition and proportion of oral *Veillonella* species in saliva of Japanese children with different oral hygiene status.

Furthermore, we determined the phylogenetic position of the unknown *Veillonella* strains evaluated by the genus-specific PCR primer set as members of the genus *Veillonella* with a phylogenetic tree.

## Methods

### Subjects

The 15 children selected to take part in the study were 6 boys and 9 girls, aged 4 to 14 years old. Participants were recruited in-person during appointments at the Dental Hospital, Health Sciences University of Hokkaido. The subjects who had a history of immunosuppression or systemic diseases (e.g. leukemia, hepatitis), or any conditions requiring antibiotic monitoring or treatment procedures (e.g. heart conditions, bone fractures), or those with mucosal lesions, previous chemotherapy, radiation therapy, or medications that can reduce the salivary flow, and those that underwent treatment with antimicrobials within the previous three months were excluded from this study.

Subjects of this study were divided into three groups based on their evaluation by the Simplified Oral Hygiene Index (OHI-s) into good, moderate, and poor oral hygiene groups, according to the criteria of Greene and Vermillion ^22^. Owing to the small number of children with poor hygiene (n=5), it was decided that 5 children would be chosen for each group. The good oral hygiene group (OHI-S score: 0–1.2) was composed of two males and three females. The moderate group (OHI-S score: 1.3–3.0) was composed of 3 males and 2 females. The poor group (OHI-S score: 3.1–6.0) was composed of 1 male and 4 females.

### Sample collection

The saliva samples were collected at the Dental Hospital, Health Sciences University of Hokkaido, Japan, over a period between 2016 and 2017. Saliva was stimulated by paraffin chewing for 1 min and was then collected into sterile plastic tubes, and transferred to an anaerobic box (Hirasawa Works, Inc., Osaka, Japan) containing 10% H _2_, 85% N _2_, 5% CO _2_. These samples (1 ml each) were transferred to 1.5-ml Eppendorf tubes, then homogenized for 1 min with a BioMasher ^®^II (Nippi, Incoporated Protein Engineering Office, Tokyo, Japan).

### Culture conditions

These homogenized saliva samples were serially diluted by 10-fold with sterile phosphate buffer saline (PBS) from 10 ^-3^ to 10 ^-7^. Aliquots (100 µl) of each diluted sample were inoculated into Bacto ^TM^ Brain Heart Infusion (BHI, Difco Laboratories, Detroit, MI, USA) supplemented with 5% (volume/volume) defibrinated sheep blood (BHI agar), hemin (10 μg/mL, Wako, Osaka, Japan), menadione (5 µg/ml, Wako), and the selective medium *Veillonella* agar ^23^. After inoculation, all media were incubated under anaerobic conditions with 10% H _2_, 85% N _2_, and 5% CO _2_ at 37°C. *Veillonella* agar was incubated for 5 days and BHI agar was incubated for 7 days. The bacterial colonies grown on BHI and *Veillonella* agar were counted as the total number of bacteria and typical *Veillonella* colonies in the saliva sample, respectively. Bacterial cells of typical *Veillonella* colonies were confirmed as gram-negative cocci with light microscopy after gram staining. Standard strains consisted of *V. atypica* ATCC 17744 ^T^, *V. denticariosi* JCM 15641 ^T^, *V. dispar* ATCC 17748 ^T^, *V. parvula* ATCC 10790 ^T^, *V. rogosa* JCM 15642 ^T^, and *V. tobetsuensis* ATCCBAA-2400 ^T^.

### DNA extraction

The genomic DNA was extracted from the isolated bacterial cells by using Insta Gene Matrix Kit (Bio-Rad Laboratories, Hercules, CA, USA). The DNA concentration determination was based on fluorescence by using a Qubit 3.0 Fluorometer. (Invitrogen, Carlsbad, CA, USA), according to the manufacturer’s protocol. Additionally, ** genomic DNA extracted from the standard strains stated above was used as positive control for PCR.

### Identification of *Veillonella* species

For the identification of *Veillonella* species at the genus level, a genus-specific PCR primer pair, Veill-rpoBF and Veill-rpoBR, were used according to the protocols described by Arif *et al.* and Mashima *et al.* ^12, 13, 24^. Strains confirmed by PCR as members of genus *Veillonella* were then subject to the one-step PCR method with the ** species-specific primers sets ATYR, DENR, DISR, PARR, ROGR, TOBR, and VF, performed according to the method reported by Mashima *et al.*, for identification at species level ^12^.

The PCR products were applied to a 2.0% agarose gel, and after electrophoresis, the gel was stained with SYBR ^®^ Safe DNA gel stain (Invitrogen).

### Phylogenetic analysis of unknown strains

For phylogenetic analysis of unknown strains, genomic DNA was also extracted from bacterial cells of unknown *Veillonella* strains showing positive PCR reaction with the genus-specific primer, but negative with the species-specific primer sets. In addition, PCR-based amplification and sequence analysis of *rpoB* were performed using the previously described primers for genus *Veillonella rpoB*-forward (5’-GTA ACA AAG GTG TCG TTT CTC G-3’) and *rpoB*-reverse (5’-GCA CCR TCA AAT ACA GGT GTA GC-3’) ^24^.

The PCR product contained DNA fragments were purified by using QIAquick ^®^ Gel Extraction Kit (Qiagen, Hilden, NW, Germany), according to the manufacturer’s instructions. The DNA concentration after purification was determined based on fluorescence using a Qubit ^®^ 3.0 Fluorometer dsDNA HS Assay Kit (Invitrogen life Technologies, Carlsbad, CA, USA. The PCR reaction was performed with 15–20 ng/µl of DNA template for cycle sequence.

Purified DNA from PCR was sequenced with an BigDye ^®^ Terminator v1.1 Cycle Sequencing kit (Thermo Fisher, Waltham, MA, USA), BigDye ^®^ Terminator 5X Sequencing Buffer (Thermo Fisher, Waltham, MA, USA), single primer 1 pM and PCR product in a final volume of 20 µl. Cycle sequencing of the purified DNA was as follows: preheating at 96°C for 1 minutes; followed by 25 cycles of denaturation at 96°C for 10 seconds and annealing with extension at 60°C for 4 minutes ^12^. Furthermore, the sequencing of PCR products were purified by using Centri-Sep column (Princeton Separations, Adelphia, NJ, USA), according to the manufacture’s instruction and resolved for the sequencing analysis.

DNA sequences were determined using an ABI PRISM 310 Genetic Analyzer (Applied Biosystem) and were aligned and connected using SEQMAN Pro from the LASERGENE program (DNASTAR). The programs MEGALIGN, which includes CLUSTALW and NJPlot were used to compare sequences and to reconstruct an evolutionary tree by the neighbour-joining method ^25^. Confidence intervals were also assessed by CLUSTALW with bootstrap analysis. Furthermore, pairwise similarity values were determined with MEGALIGN in the LASERGENE program. The *rpoB* sequences of the unknown *Veillonella* strains were aligned against the sequence of the established *Veillonella* species retrieved from GenBank. Unipro UGENE could be use as free alternative for both sequencing and pairwise similarity values.

### Ethical considerations

All subjects and their parents were made aware of the objectives and procedures of the study and parents of participants provided written informed consent. This study was conducted with the approval of The Ethics Committee of the Health Sciences University of Hokkaido, Japan, under process number of 2016-015

## Results

### Colony numbers

The average number of colony forming units (CFU)/ml of all bacteria on BHI agar increased with decreased oral hygiene: 1.38E+08, 2.2E+08 and 4.48E+09 in the good, moderate and poor groups, respectively. Raw CFU data are available on Figshare ^26^.

### Species identification

The phenotypic characteristics of *Veillonella* colonies on the selective medium were 2–4 mm in diameter, and slightly domed in shape with an entire edge, opaque, and greyish white. They were composed of small, gram-negative coccal cells, mainly existing as single cells but with short chains visible. In the good oral hygiene group, a mean number of 1.70E+06 CFU/ml (median, 1.20E+06 CFU/ml) were identified as the genus *Veillonella*, with 12.3% *V. atypica*, 19.3% *V. dispar*, 10.5% *V. parvula*, 49.1% *V. rogosae*, and 8.8% unknown species ( Table 1). In the moderate group, 2.08E+07 CFU/ml with median 2.00E+06 were identified as the genus *Veillonella*, ** with 6.2% *V. atypica*, 29.6% *V. dispar*, 12.3% *V. parvula*, 44.4% *V. rogosae*, and 7.4% unknown species ( Table 2). Meanwhile, in the poor oral hygiene group, 4.48E+09 CFU/ml with median 2.20E+06 were identified as the genus *Veillonella*, with 7.3% *V. atypica*, 12.2% *V. dispar*, 31.7% *V. parvula*, 34.1% *V. rogosae*, and 14.6% unknown species proportions ( Table 3).

**Table 1.** Ratio of the number of isolates of each species to the total number of *Veillonella* isolate in saliva from the good oral hygiene group. The colony-forming units (CFU) of all anaerobic bacteria on brain heart infusion agar and *Veillonella* strains on *Veillonella* agar (detection limit <0.1% of the total count). The total of *Veillonella* isolates identified by the *Veillonella* genus-specific PCR primer. Individual species as a percentage of the number of isolates identified by one-step PCR with the species-specific primer sets for each subject ( *n* = 5) from saliva of the good oral hygiene group.

Subject			Total number		Isolated *Veillonella* species							
Name	Age	Sex	All bacteria	*Veillonella* spp.	Total number	*V.* *atypica*	*V.* *denticaruisi*	*V.* *dispar*	*V.* *parvula*	*V.* *rogosae*	*V.* *tobetsuensis*	Unknown
			CFU/mL	CFU/mL	(100%)	(%)	(%)	(%)	(%)	(%)	(%)	(%)
S8	13	F	7.60E+07	3.00E+05	3	0	0	0	1 (33.3)	2 (66.7)	0	0
S9	6	M	2.04E+08	5.00E+06	5	2 (40.0)	0	0	0	1 (20.0)	0	2 (40.0)
S12	6	F	3.52E+08	1.20E+06	12	0	0	4 (33.3)	0	8 (66.7)	0	0
S25	6	F	2.70E+07	2.00E+06	20	2 (10.0)	0	3 (15.0)	4 (20.0)	9 (45.0)	0	2 (10.0)
S28	8	M	3.20E+07	1.70E+02	17	3 (17.6)	0	4 (23.5)	1 (5.9)	8 (47.1)	0	1 (5.9)

**Table 2.** Ratio of the number of isolates of each species to the total number of *Veillonella* isolate in saliva from the moderate oral hygiene group. The colony-forming units (CFU) of all anaerobic bacteria on the brain heart infusion agar and *Veillonella* strains on *Veillonella* agar (detection limit <0.1% of the total count). The total of *Veillonella* isolates identified by the *Veillonella* genus-specific PCR primer. Individual species as a percentage of the number of isolates identified by one-step PCR with the species-specific primer sets for each subject ( *n* = 5) from saliva of the moderate oral hygiene group.

Subject			Total number		Isolated *Veillonella* species							
Name	Age	Sex	All bacteria	*Veillonella* spp.	Total number	*V.* *atypica*	*V.* *denticaruisi*	*V.* *dispar*	*V.* *parvula*	*V.* *rogosae*	*V.* *tobetsuensis*	Unknown
			CFU/mL	CFU/mL	(100%)	(%)	(%)	(%)	(%)	(%)	(%)	(%)
S1	9	M	1.04E+08	2.00E+06	20	3 (15.0)	0	11 (55.0)	2 (10.0)	4 (20.0)	0	0
S3	4	M	5.30E+08	1.20E+07	12	0	0	3 (25.0)	1 (8.3)	4 (33.3)	0	4 (33.3)
S10	7	M	4.16E+08	9.00E+07	9	0	0	0	5 (55.6)	3 (33.3)	0	1 (11.1)
S29	6	F	4.46E+07	2.00E+05	20	2 (10.0)	0	5 (25.0)	2 (10.0)	10 (50.0)	0	1 (5.0)
S32	14	F	3.90E+06	2.00E+04	20	0	0	5 (25.0)	0 (0.0)	15 (75.0)	0	0

**Table 3.** Ratio of the number of isolates of each species to the total number of *Veillonella* isolated in saliva from the poor oral hygiene group. The colony-forming units (CFU) of all anaerobic bacteria on the brain heart infusion agar and *Veillonella* strains on *Veillonella* agar (detection limit <0.1% of the total count). The total of *Veillonella* isolates identified by the *Veillonella* genus-specific PCR primer. Individual species as a percentage of the number of isolates identified by one-step PCR with the species-specific primer sets for each subject ( *n*=5) from saliva of the poor oral hygiene group.

Subject			Total number		Isolated *Veillonella* species							
Name	Age	Sex	All bacteria	*Veillonella* spp.	Total number	*V.* *atypica*	*V.* *denticaruisi*	*V.* *dispar*	*V.* *parvula*	*V.* *rogosae*	*V.* *tobetsuensis*	Unknown
			CFU/mL	CFU/mL	(100%)	(%)	(%)	(%)	(%)	(%)	(%)	(%)
S15	7	M	4.00E+09	1.40E+08	14	0	0	3 (21.4)	1 (7.1)	8 (57.1)	0	2 (14.3)
S16	9	F	4.64E+09	3.00E+08	3	0	0	0	0	3 (100.0)	0	0
S17	10	F	7.84E+09	5.00E+08	5	3 (60.0)	0	0	0	1 (20.0)	0	1 (20.0)
S21	8	F	1.50E+09	1.00E+08	10	0	0	0	8 (80.0)	0	0	2 (20.0)
S30	8	F	4.41E+09	9.00E+08	9	0	0	2 (22.2)	4 (44.4)	2 (22.2)	0	1 (11.1)

**Figure 1.**
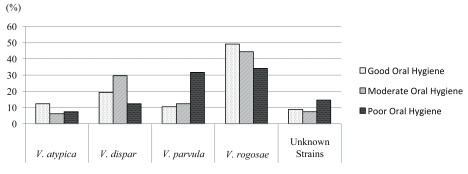
Total isolated number of each *Veillonella* species isolated. Data expressed as percentage of total the total isolated number as *Veillonella* in samples from saliva in good, moderate and poor oral hygiene groups.

As shown in the results, *V. rogosae* was found as the predominant species in the saliva samples of all oral hygiene groups. However, *V. denticariosi* and *V. tobetsuensis* were not found in all oral hygiene groups ( Table 1– Table 3). Figure 1 shows the per cent ratio of the total number of strains of each species to the total number of *Veillonella* isolates from saliva samples of the good, moderate, and poor oral hygiene groups.

### Strain characteristics

**Figure 2.**
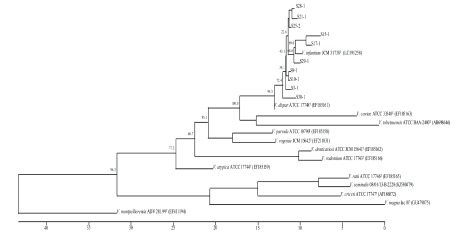
Neighbor-joining tree showing the relationship between the 10 unknown *Veillonella* strains and the type strains of established species of genus *Veillonella*, with accession numbers. Bootstrap values were indicated at corresponding nodes. The scale bar indicated the number of nucleotide substitutions per 100 residues.

In this study, 179 strains were identified as *Veillonella* strains, and 162 strains were identified as *V. atypica*, *V. dispar*, *V. parvula* or *V. rogosae*. However, 17 (9.5%) of 179 strains failed to be classified as any of the known oral *Veillonella* species, thus, they were defined as unknown species. Of the 17 unknown *Veillonella* strains 10 (S3-1, S9-1, S10-1, S15-1, S17-1, S21-1, S25-2, S28-1, S29-1 and S30-1) were selected as representative strains from different hygiene groups for phylogenetic analysis. After determination of the *rpoB* sequences of these 10 strains, these sequences were aligned toward to the sequences of *Veillonella* type strains retrieved from GenBank. The evolutionary tree produced by analysing the type strains of the 14 *Veillonella* species and the 10 unknown strains is shown in the Figure 2. According to this data, the most closely related species was *V. infantium*, although the 10 unknown strains formed three clusters. The DNA sequence similarity of the 10 unknown *Veillonella* strains to *V infantium* JCM 31738 ^T^ (LC191258) ranged from 97.1 to 99.7%.

## Discussion

It was previously reported that a higher number of anaerobic bacteria was detected on BHI agar in saliva from Thai children with poor oral hygiene than those with good and moderate oral hygiene ^20^. This prior study demonstrated that oral *Veillonella* isolates were detected at a twofold higher frequency in the saliva of Thai children with poor rather than good or moderate oral hygiene ^20^. Here, it was demonstrated that the number of anaerobic bacteria on BHI agar and *Veillonella* species on the selective medium increased in saliva of Japanese children with worsening oral hygiene status. Therefore, the detection level of anaerobic bacterial strains and oral *Veillonella* strains in saliva from Japanese children with good, moderate and poor oral hygiene status was similar to that from Thai children.

Using the Illumina MiSeq platform, Mashima *et al.* demonstrated that *Streptococcus* and *Veillonella* species were the predominant bacterial species in the saliva microbiome of Thai children, but that the proportion of *Streptococcus* decreased while that of *Veillonella* increased with poor oral hygiene status ^5^. They also found that *Veillonella* species were detected predominantly in the tongue microbiome of Thai children with poor oral hygiene status compared to those with good or moderate oral hygiene status ^5^. Taken together with the results of the present study, it is possible that *Veillonella* species could be a biomarker of oral hygiene status for Thai and Japanese children.

This study demonstrated that *V. rogosae* was the predominant species detected in all groups of Japanese children ( Figure 1). Beighton *et al.* reported *V. rogosae* as one of the predominant *Veillonella* species in tongue biofilms of healthy adults in the UK ^24^. A previous study also showed that *V. rogosae* was the predominant *Veillonella* species in tongue biofilms of the children in Thailand ^12^. Recently, Theodorea *et al.* isolated 1,609 *Veillonella* strains from saliva samples of Thai children divided into three groups: good, moderate and poor oral hygiene status ^20^. Then, 1,442 of 1,609 strains were detected by the one-step PCR method with the species-specific primer sets for *V. atypica*, *V. denticariosi*, *V. dispar*, *V. parvula*, *V. rogosae*, or *V. tobetsuensis.* They reported that *V. rogosae* was the predominant species detected in all groups ^20^. These results of the previous and present studies indicate that *V. rogosae* is the predominant oral *Veillonella* species in the human saliva and tongue biofilm.

Furthermore, this study showed that the detection rate of *V. rogosae* decreased as oral hygiene quality decreased: 49.1%, 44.4%, and 34.1% in the good, moderate, and poor oral hygiene groups, respectively ( Figure 1). Similar results were obtained from saliva samples of Thai children were also reported by Theodorea *et al.* ^20^. Based on these results, it was demonstrated that the detection rate of *V. rogosae* decreased with aggravation of oral hygiene status in Japanese and Thai children. Additionally, Arif *et al.* detected *V. rogosae* only in carious-free lesions of dental plaques ^13^. All these data suggest that a human oral cavity with good hygiene status is suitable habitat for *V. rogosae*.

Conversely, the detection rate of *V. parvula* in the poor (31.7%) oral hygiene was significantly higher than that in the good (10.5%) and moderate (12.3%) oral hygiene groups, in this study with Japanese children. This result is conformed with data from another study, in which *V. parvula* was most frequently detected in saliva of Thai children with poor oral hygiene status ^20^. Previously, it was also reported that *V. parvula* was frequently detected in periodontal pockets ^27^ and active carious-lesions ^28^. These data suggest that oral cavities with poor hygiene status are suitable environments for *V. parvula*.

In this study, 179 strains were isolated members of the genus *Veillonella* from saliva of 15 Japanese children, *V. denticariosi* and *V. tobetsuensis* were not found in any samples. In the case of saliva samples from Thai children, the detection rate of *V. denticariosi* (0.4%) and *V. tobetsuensis* (1.7%) were very low. In this study 1,609 *Veillonella* strains were isolated from 107 Thai children ^20^. Similarly, it was reported that *V. denticariosi* was not detected in any of the tongue biofilms of Thai children ^12^, and *V. denticariosi* was detected in tongue biofilm of only one young Japanese adult ^17^. Therefore, *V. denticariosi* may be the least prevalent oral *Veillonella* species in the saliva and tongue microbiome. On the other hand, *V. tobetsuensis*, ** was not detected in saliva from Thai children with good oral hygiene status. However, the detection rate of *V. tobetsuensis* was 14.3% and 17.8% in the saliva of Thai children with moderate and poor oral hygiene, respectively (20). Similarly, it was demonstrated that the prevalence of *V. tobetsuensis* ranged from 7.6% to 20.0% in tongue biofilm samples from Japanese adults ^16^. Therefore, these data suggest that *V. tobetsuensis* may be potential to co-occur with other *Veillonella* species in saliva and tongue biofilms.

In the present study with saliva samples of Japanese children, 17 (9.5%) of 179 strains confirmed as member of genus *Veillonella* were not belong to any established *Veillonella* species as unknown species. Theodorea *et al.*, also reported that 167 (10.4%) of 1,609 *Veillonella* isolates from saliva of Thai children could not be assigned to any species of the genus *Veillonella* ^20^. Furthermore, it was reported that 43 (9.7%) of the 442 *Veillonella* isolates from periodontal pockets and gingival sulcus could not be identified as any of the known *Veillonella* species. These data may indicate that other novel *Veillonella* species inhabit human oral cavities, although only six species were detected as oral *Veillonella* species up to this point. Further phylogenetic studies are needed to evaluate the possibilities of novel *Veillonella* species.

In 2018, Mashima *et al*. ^11^ proposed *V. infantium* as a novel species isolated from saliva of Thai children, representing a seventh oral *Veillonella* species. Therefore, for phylogenetic analysis of the unknown *Veillonella* strains isolated in this study, the *rpoB* sequences of type strains of the established *Veillonella* species, including *V. infantium* JCM 31738 ^T^, were examined. Consequently, 10 unknown *Veillonella* strains analysed in this study formed three clusters distinct from *V. dispar*, the most closely related species was *V. infantium.* Further studies are required to assign these strains most accurately. ****

In conclusions, this is the first study to identify oral *Veillonella* at the species level in the saliva of Japanese children divided into three oral hygiene status groups: good, moderate and poor group. Although *V. denticariosi* and *V. tobetsuensis* were not found in any groups in this study because of small number of subjects, the distribution and frequency of *V. atypica*, *V. dispar*, *V. parvula* and *V. rogosae*, were mostly the same as those in the saliva from Thai children divided into the aforementioned oral hygiene status groups. Additionally, the results of this study demonstrate that changes in the ratio of some *Veillonella* species, such as an increase of *V. parvula* and decrease of *V. rogosae* in those with poor oral hygiene, can be a useful indicator of oral hygiene status, as with results obtained in the study of saliva taken from Thai children. The present study also showed that approximately 10% of the isolated *Veillonella* strains were not classified to any *Veillonella* species, and that they will be assigned to *V. infantium* or novel *Veillonella* species after further studies.

## Data availability

16S rRNA sequences of the 10 unknown *Veillonella* strains are available from GenBank, accession numbers: LC467206 (S9-1), LC467207 (S28-1), LC467208 (S25-1), LC467209 (S17-1), LC467210 (S15-1), LC467211 (S29-1), LC467212 (S10-1), LC467213 (S30-1), LC467214 (S21-1), LC467215 (S3-1).

Figshare: Raw Data Saliva Japanese Children.xlsx. https://doi.org/10.6084/m9.figshare.7856657.v1 ^26^.

This project contains the number of colony-forming units and total number of *Veillonella* strains isolated from each child.

Data on Figshare are available under the terms of the Creative Commons Attribution 4.0 International license (CC-BY 4.0).
