# Supplementary material for: Identification and phylogenetic analysis of oral Veillonella species isolated from the saliva of Japanese children
Source: F1000Res. 2019 Sep 13;8:616. Originally published 2019 May 3. [Version 5] doi: 10.12688/f1000research.18506.5 (PMC6688723; doi:10.12688/f1000research.18506.5)
Supplement: Peer review history

# Peer review: Reviewer response for version 5

**DOI:** 10.5256/f1000research.22657.r53912

**Published:** 2019-09-16

**Authors:** Takuichi Sato

**Affiliations:** 1 Division of Clinical Chemistry, Department of Medical Technology, Niigata University Graduate School of Health Sciences, Niigata, Japan

OK

I confirm that I have read this submission and believe that I have an appropriate level of expertise to confirm that it is of an acceptable scientific standard.

# Peer review: Reviewer response for version 4

**DOI:** 10.5256/f1000research.22192.r52215

**Published:** 2019-08-08

**Authors:** Takuichi Sato

**Affiliations:** 1 Division of Clinical Chemistry, Department of Medical Technology, Niigata University Graduate School of Health Sciences, Niigata, Japan

I have recognized and agreed with the revised parts in the Introduction.

I confirm that I have read this submission and believe that I have an appropriate level of expertise to confirm that it is of an acceptable scientific standard.

# Peer review: Reviewer response for version 3

**DOI:** 10.5256/f1000research.21912.r51348

**Published:** 2019-08-01

**Authors:** Takuichi Sato

**Affiliations:** 1 Division of Clinical Chemistry, Department of Medical Technology, Niigata University Graduate School of Health Sciences, Niigata, Japan

[Suggestions]

 I recognized the revised parts in the Abstract (Background) as follows;

 "However, the study regarding the identification and distribution of oral Veillonella are limited. The oral Veillonella community may affected by the differences in geographical location, age, diet, lifestyle, socio-economic status and oral hygiene status."

 I think that the above-mentioned parts should be included in the Introduction, but I was unable to find the revised parts. The authors need to revise the Introduction, like as in the Abstract.

 [Typographical errors]

 Introduction (6th paragraph)

 "difficult to idnetify" should read "difficult to identify".

I confirm that I have read this submission and believe that I have an appropriate level of expertise to confirm that it is of an acceptable scientific standard, however I have significant reservations, as outlined above.

# Response

**Published:** 2019-08-03

**Authors:** Ariadna Djais

**Affiliations:** University of Indonesia, Indonesia

The authors would like to thank the area editor and the reviewers for their precious time and valuable comments. We have carefully addressed all the comments.

**Reviewer: Takuichi Sato,** Division of Clinical Chemistry, Department of Medical Technology, Niigata University Graduate School of Health Sciences, Niigata, Japan [Suggestions]

I recognized the revised parts in the Abstract (Background) as follows;

"However, the study regarding the identification and distribution of oral Veillonella are limited. The oral Veillonella community may affected by the differences in geographical location, age, diet, lifestyle, socio-economic status and oral hygiene status."

I think that the above-mentioned parts should be included in the Introduction, but I was unable to find the revised parts. The authors need to revise the Introduction, like as in the Abstract.

[Typographical errors]

Introduction (6th paragraph)

"difficult to idnetify" should read "difficult to identify".

**Authors: **Thank you very much for this direction.

1. We have changed the wording of the relevant in the Introduction 6th Paragraph, L 11, like as in the Abstract.
2. [Typographical errors] Introduction (6th paragraph) "difficult to idnetify" into "difficult to identify".
3. Introduction 7th Paragraph, L 2 was removed because in this study we are focused on demonstrating that the composition and proportion of oral *Veillonella* species in the saliva of Japanese children is correlated with different oral hygiene status.

The revised version of this paper has been submitted.

Thank you for taking the time and energy to help us improve this manuscript. We hope that you will find that these revisions rise to your expectation.

# Peer review: Reviewer response for version 2

**DOI:** 10.5256/f1000research.21331.r50157

**Published:** 2019-06-28

**Authors:** Takuichi Sato

**Affiliations:** 1 Division of Clinical Chemistry, Department of Medical Technology, Niigata University Graduate School of Health Sciences, Niigata, Japan

The authors have described the following in the Response, but I am unable to find the differences in the abstract (and/or introduction).

 "We have changed the wording of the relevant in the abstract to express the reason why did we want to know the detection rate and distribution of oral *Veillonella *species in Japan. The reasons are the study regarding the identification and distribution of oral *Veillonella* are limited; also the oral *Veillonella* community may affected by the differences in geographical location, age, diet, lifestyle, socio-economic status and oral hygiene status."

I confirm that I have read this submission and believe that I have an appropriate level of expertise to confirm that it is of an acceptable scientific standard, however I have significant reservations, as outlined above.

# Peer review: Reviewer response for version 2

**DOI:** 10.5256/f1000research.21331.r50156

**Published:** 2019-06-27

**Authors:** Juni Handajani

**Affiliations:** 1 Department of Oral Biology, Faculty of Dentistry, Gadjah Mada University, Yogyakarta, Indonesia

Thank you for the revision. I now understand the analysis statistic correctly, no more changes are required and I approved the article.

I confirm that I have read this submission and believe that I have an appropriate level of expertise to confirm that it is of an acceptable scientific standard.

# Peer review: Reviewer response for version 1

**DOI:** 10.5256/f1000research.20250.r48113

**Published:** 2019-05-23

**Authors:** Takuichi Sato

**Affiliations:** 1 Division of Clinical Chemistry, Department of Medical Technology, Niigata University Graduate School of Health Sciences, Niigata, Japan

**Suggestions:**

Abstract and Introduction:

 I think that the manuscript, in the Abstract and Introduction, needs to express rationales of this study in more detail, such as, why did the authors want to know the detection rate and distribution of oral *Veillonella* species in saliva of Japanese children? Why did the authors want to compare the results with those of Thai children?

Figure 1 and Tables 1-3:

 I think that the data in Figure 1 and Tables 1-3 are overlapping, and Tables 1-3 could be deleted from the manuscript, because the authors mainly stated the total and the mean (proportions) of the isolates of *Veillonella* species in the Results and Discussion.

Minor:

 The authors should check the meaning of the phrase "although":

 P. 7, Discussion: in the 6th paragraph

 L. 1-2: "although 179 strains were isolated members of the genus *Veillonella* from saliva of 15 Japanese children, …".

 P. 7, Discussion: in the 6th paragraph

 L. 6-7: "although in this study 1,609 *Veillonella* strains were isolated from 107 Thai children".

 P. 8, Discussion: in the 8th paragraph

 L. 7-8: "although 10 unknown *Veillonella* strains analysed in this study formed three clusters distinct from *V. dispar*, …".

Typographical errors;

 P. 4, Results: Species identification

 L. 6: "numberof" should read "number of". (Put a space in between.)

 P. 7, Discussion: in the 5th paragraph

 L. 4: "conforms" should read "conformed".

I confirm that I have read this submission and believe that I have an appropriate level of expertise to confirm that it is of an acceptable scientific standard, however I have significant reservations, as outlined above.

# Response

**Published:** 2019-05-24

**Authors:** Ariadna Djais

**Affiliations:** University of Indonesia, Indonesia

Abstract and Introduction:

I think that the manuscript, in the Abstract and Introduction, needs to express rationales of this study in more detail, such as, why did the authors want to know the detection rate and distribution of oral *Veillonella* species in saliva of Japanese children? Why did the authors want to compare the results with those of Thai children?

Response: Thank you very much for this direction. We have changed the wording of the relevant in the abstract to express the reason why did we want to know the detection rate and distribution of oral *Veillonella *species in Japan. The reasons are the study regarding the identification and distribution of oral *Veillonella* are limited; also the oral *Veillonella* community may affected by the differences in geographical location, age, diet, lifestyle, socio-economic status and oral hygiene status.

Figure 1 and Tables 1-3:

I think that the data in Figure 1 and Tables 1-3 are overlapping, and Tables 1-3 could be deleted from the manuscript, because the authors mainly stated the total and the mean (proportions) of the isolates of *Veillonella* species in the Results and Discussion.

Response: Thanks for raising this important point. However, we consider to state

- the Table 1-3 as Ratio of the number of isolates of each species from each subject.
- Figure 1 was indicated to express the Total isolated number of each *Veillonella* species from each group of oral hygiene.

Minor:

The authors should check the meaning of the phrase "although":

P. 7, Discussion: in the 6th paragraph

L. 1-2: "although 179 strains were isolated members of the genus *Veillonella *from saliva of 15 Japanese children, …".

Response: the word of “although” has been removed

P. 7, Discussion: in the 6th paragraph

L. 6-7: "although in this study 1,609 *Veillonella* strains were isolated from 107 Thai children".

Response: the word of “although” has been removed

P. 8, Discussion: in the 8th paragraph

L. 7-8: "although 10 unknown *Veillonella *strains analysed in this study formed three clusters distinct from *V. dispar*, …".

Response: the word of “although” has been removed

Typographical errors;

P. 4, Results: Species identification

L. 6: "numberof" should read "number of". (Put a space in between.)

Response: the word of “numberof” has been revised to “number of”

P. 7, Discussion: in the 5th paragraph

L. 4: "conforms" should read "conformed".

Response: the word of “conforms” has been revised to “conformed”

Thank you for taking the time and energy to help us improve this manuscript. We hope that you will find these revision rise to your expectation.

# Peer review: Reviewer response for version 1

**DOI:** 10.5256/f1000research.20250.r48110

**Published:** 2019-05-20

**Authors:** Juni Handajani

**Affiliations:** 1 Department of Oral Biology, Faculty of Dentistry, Gadjah Mada University, Yogyakarta, Indonesia

This study is good for identification and phylogenetic analysis of oral *Veillonella* species isolated from saliva samples of children in Japan. The aim of this study was to analyze the composition and proportion of oral *Veillonella* species in the saliva of Japanese children compared to previous studies in Thailand.

 The manuscript is certainly well written but I have some major concerns on the data analysis:

1. The number of samples in this study was fewer (15 children) compared to the number of samples in the previous study from Thailand (107 children). It is suggested that the analysis uses proportions so that it can describe the oral *Veillonella* species according to the number of samples.
2. To find out the comparison of the results of this study with the results of a previous study from Thailand, a correlation analysis is suggested.
3. It is also necessary to add the results of a correlation analysis between the results of *Veillonella*'s oral identification and oral health status.

 

 Minor comment:

1. Some references used are older than 10 years. I suggest to use current references from at least the last 10 years.

I confirm that I have read this submission and believe that I have an appropriate level of expertise to confirm that it is of an acceptable scientific standard, however I have significant reservations, as outlined above.

# Response

**Published:** 2019-05-24

**Authors:** Ariadna Djais

**Affiliations:** University of Indonesia, Indonesia

This study is good for identification and phylogenetic analysis of oral Veillonella species isolated from saliva samples of children in Japan. The aim of this study was to analyze the composition and proportion of oral *Veillonella* species in the saliva of Japanese children compared to previous studies in Thailand.

Response: Thank you for your valuable comment. The aim of this study was focus to analyze the composition and proportion of oral *Veillonella* species in the saliva of Japanese children. Furthermore, we compared the results with some reports such as in Thailand. The revise version has been submitted.

The manuscript is certainly well written but I have some major concerns on the data analysis:

1. The number of samples in this study was fewer (15 children) compared to the number of samples in the previous study from Thailand (107 children). It is suggested that the analysis uses proportions so that it can describe the oral *Veillonella* species according to the number of samples.

Response: These children visited to the Dental Hospital, Health Sciences University of Hokkaido for dental examination, over a period between 2016 and 2017. Based on the evaluation by the Simplified Oral Hygiene Index, they were divided into three groups, good, moderate and poor. But, among many children, only five children had poor oral hygiene status. Therefore, we used these five children as poor group. And we have selected five children from good and moderate groups, without distinction. As the results, the total subject : 15 subjects.

1. To find out the comparison of the results of this study with the results of a previous study from Thailand, a correlation analysis is suggested.
2. It is also necessary to add the results of a correlation analysis between the results of Veillonella's oral identification and oral health status.

Response: Thank you for another good point at No. 2 and No.3. The research of oral *Veillonella* were very limited. The analysis was use in this study : descriptive analysis with mean value to determine the distribution and proportion of oral *Veillonella*, then we compare the characteristic of oral veillonella distribution with the previous study.

Minor comment:

1. Some references used are older than 10 years. I suggest to use current references from at least the last 10 years.

 

Response: As we mentioned before, that the research of oral *Veillonella* were very limited. Therefore, some of references were used older that 10 years.

Thank you for taking the time and energy to help us improve this manuscript. We hope that you will find these revision rise to your expectation.
